# The role of the law in reducing tuberculosis transmission in Botswana, South Africa and Zambia

**DOI:** 10.2471/BLT.15.156927

**Published:** 2015-02-12

**Authors:** Andre R Verani, Courtney N Emerson, Philip Lederer, Ginny Lipke, Nathan Kapata, Samson Lanje, Annatjie C Peters, Isaac Zulu, Barbara J Marston, Bess Miller

**Affiliations:** a Division of Global HIV and TB, Centers for Disease Control and Prevention, 1600 Clifton Road, Atlanta, Mailbox E-39, GA 30329, United States of America (USA).; b National Tuberculosis and Leprosy Control Programme, Ministry of Health, Lusaka, Zambia.; c United States Centers for Disease Control and Prevention, Gaborone, Botswana.; d Jhpiego South Africa, MDR TB Partnership, Pretoria, South Africa.; e Division of Global Health Protection, Centers for Disease Control and Prevention, Atlanta, USA; f Independent Global Public Health Consultant, Atlanta, USA.

## Abstract

**Objective:**

To determine whether laws and regulations in Botswana, South Africa and Zambia – three countries with a high tuberculosis and HIV infection burden – address elements of the World Health Organization (WHO) policy on tuberculosis infection control.

**Methods:**

An online desk review of laws and regulations that address six selected elements of the WHO policy on tuberculosis infection control in the three countries was conducted in November 2015 using publicly available domestic legal databases. The six elements covered: (i) national policy and legal framework; (ii) health facility design, construction and use; (iii) tuberculosis disease surveillance among health workers; (iv) patients’ and health workers’ rights; (v) monitoring of infection control measures; and (vi) relevant research.

**Findings:**

The six elements were found to be adequately addressed in the three countries’ laws and regulations. In all three, tuberculosis case-reporting is required, as is tuberculosis surveillance among health workers. Each country’s legal and regulatory framework also addresses the need to respect individuals’ rights and privacy while safeguarding public health. These laws and regulations create a strong foundation for tuberculosis infection control. Although the legal and regulatory frameworks thoroughly address tuberculosis infection control, their dissemination, implementation and enforcement were not assessed, nor was their impact on public health.

**Conclusion:**

Laws and regulations in Botswana, South Africa and Zambia address all six selected elements of the WHO policy on tuberculosis infection control. However, the lack of data on their implementation is a limitation. Future research should assess the implementation and public health impact of laws and regulations.

## Résumé

**Objectif:**

Déterminer si les lois et les règlements de l'Afrique du Sud, du Botswana et de la Zambie – trois pays ayant une forte charge de morbidité liée à la tuberculose et au VIH – prennent en compte les éléments de la politique de lutte contre la tuberculose de l'Organisation mondiale de la Santé (OMS).

**Méthodes:**

Un examen en ligne des lois et règlements tenant compte de six éléments donnés de la politique de lutte contre la tuberculose de l'OMS dans ces trois pays a été réalisé en novembre 2015 à partir de bases de données juridiques nationales accessibles au public. Ces six éléments portaient sur: (i) le cadre politique et juridique national; (ii) la conception, la construction et l'utilisation des établissements de santé; (iii) la surveillance de la tuberculose chez les professionnels de santé; (iv) les droits des patients et des professionnels de santé; (v) le suivi des mesures de lutte contre la maladie; et (vi) les activités de recherche pertinentes.

**Résultats:**

Il est apparu que ces six éléments étaient bien pris en compte dans les lois et règlements des trois pays. Dans ces trois pays, le signalement des cas de tuberculose ainsi que la surveillance de la maladie chez les professionnels de santé sont obligatoires. Le cadre juridique et règlementaire de chaque pays mentionne également la nécessité de respecter les droits et l'intimité des personnes tout en protégeant la santé publique. Ces lois et règlements constituent une base solide de lutte contre la tuberculose. Cependant, bien que ces cadres juridiques et règlementaires intègrent dûment la lutte contre la tuberculose, leur diffusion, leur mise en œuvre et leur application n'ont pas été évaluées, pas plus que leur impact sur la santé publique.

**Conclusion:**

Les lois et les règlements de l'Afrique du Sud, du Botswana et de la Zambie tiennent compte des six éléments sélectionnés de la politique de lutte contre la tuberculose de l'OMS. Cependant, le manque de données sur leur mise en œuvre est regrettable. Les recherches futures devraient évaluer la mise en œuvre et l'impact sur la santé publique de ces lois et règlements.

## Resumen

**Objetivo:**

Determinar si la legislación y normativas de Botswana, Sudáfrica y Zambia (tres países con altas tasas de contagio de la tuberculosis y el VIH) abordan elementos de la política de la Organización Mundial de la Salud (OMS) sobre el control de contagio de la tuberculosis.

**Métodos:**

En noviembre de 2015 se realizó un estudio preliminar en línea de la legislación y normativas que tratan los seis elementos seleccionados de la política de la OMS sobre el control de contagio de la tuberculosis en los tres países utilizando bases de datos legales nacionales que se encuentran a disposición pública. Los seis elementos abarcaban: (i) política nacional y marco legal; (ii) diseño, construcción y utilización de instalaciones sanitarias; (iii) control de la tuberculosis entre trabajadores sanitarios; (iv) derechos de los pacientes y trabajadores sanitarios; (v) supervisión de las medidas de control de contagios; e (vi) investigación pertinente.

**Resultados:**

Se consideró que la legislación y normativas de los tres países abordaban adecuadamente los seis elementos. En los tres países es necesario tanto informar de los casos de tuberculosis como controlar esta enfermedad entre trabajadores sanitarios. El marco legal y normativo de cada país también trata la necesidad de respetar los derechos y privacidad de los individuos al mismo tiempo que se salvaguarda la salud pública. Estas leyes y normativas crean una base sólida para el control del contagio de la tuberculosis. A pesar de que los marcos legales y normativos abordan completamente el control del contagio de la tuberculosis, no se evaluó su divulgación, implementación y aplicación, ni tampoco sus consecuencias para la salud pública.

**Conclusión:**

La legislación y normativas de Botswana, Sudáfrica y Zambia abordan los seis elementos de la política de la OMS sobre el control del contagio de la tuberculosis. No obstante, la carencia de datos sobre su implementación supone una limitación. Las futuras investigaciones deberán evaluar la implementación y las consecuencias para la salud pública de las leyes y normativas.

## ملخص

**الغرض:**

تحديد ما إذا كانت القوانين واللوائح التنظيمية في بوتسوانا وجنوب أفريقيا وزامبيا – ثلاث بلدان تواجه عبئًا مرتفعًا نتيجة انتشار عدوى السل وفيروس العوز المناعي البشري – تتناول العناصر الواردة في سياسة منظمة الصحة العالمية مكافحة عدوى السل.

**الأسلوب:**

أُجريت مراجعة وتدقيق مكتبي على شبكة الإنترنت للقوانين واللوائح التنظيمية التي تتناول ستة عناصر محددة واردة في سياسة منظمة الصحة العالمية بشأن مكافحة عدوى السل في البلدان الثلاثة في نوفمبر/تشرين الثاني 2015 باستخدام قواعد البيانات القانونية المحلية المتاحة لعامة الشعب. وقامت العناصر الستة بتغطية ما يلي: (1) السياسة الوطنية والإطار القانوني؛ و(2) تصميم المرافق الصحية وإنشائها واستخدامها؛ و(3) مراقبة مرض السل بين العاملين في مجال الصحة؛ و(4) حقوق المرضى والعاملين في مجال الصحة؛ و(5) مراقبة إجراءات مكافحة العدوى؛ و(6) البحوث ذات الصلة.

**النتائج:**

لقد وُجد أن العناصر الستة تم تناولها بشكلٍ كافٍ في القوانين واللوائح التنظيمية السائدة في البلدان الثلاثة. في البلدان الثلاثة، يلزم الإبلاغ عن حالة الإصابة بمرض السل، كما أنه يلزم مراقبة مرض السل بين العاملين في مجال الصحة. يتناول الإطار القانوني والتنظيمي لكل دولة أيضًا الحاجة إلى احترام حقوق وخصوصية الأفراد في أثناء حماية الصحة العامة. تؤدي هذه القوانين واللوائح التنظيمية إلى إنشاء أساس قوي لمكافحة عدوى السل. على الرغم من أن الأطر القانونية والتنظيمية تتناول قضية مكافحة عدوى السل بشكل شامل، لم يتم تقييم طريقة نشرها وتنفيذها وإنفاذها، ولم يتم تقييم تأثيرها على الصحة العامة.

**الاستنتاج:**

تتناول القوانين واللوائح التنظيمية في بوتسوانا وجنوب أفريقيا وزامبيا جميع العناصر الستة المحددة والواردة في سياسة منظمة الصحة العالمية بشأن مكافحة عدوى السل. إلا أن نقص البيانات المتعلقة بطريقة تنفيذها يمثل أحد القيود المفروضة. ينبغي أن يتم تقييم طريقة تنفيذ القوانين واللوائح التنظيمية وتأثيرها على الصحة في البحوث التي يتم إجراؤها في المستقبل.

## 摘要

**目的:**

旨在确定在结核病和 HIV 感染高发的博茨瓦纳、南非和赞比亚三个国家，法律法规是否论述了世界卫生组织 (WHO) 关于结核病感染控制政策的原则。

**方法:**

2015 年利用公开的国内法律数据库，展开一次法律法规的在线案头审查，其论述了 WHO 关于此三个国家的结核病感染控制政策的六项选定原则。此六项原则包括：(i) 国家政策和法律框架；(ii) 卫生设备设计、建筑和使用；(iii) 健康工作者的结核病疾病监控；(iv) 患者和健康工作者的权利；(v) 监控感染控制措施；以及 (vi) 相关研究。

**结果:**

此三个国家的法律法规充分论述了六项原则。在这三个国家，需要结核病案例报告，以及健康工作者之间的结核病监控。每个国家的法律法规框架同样满足保卫公共健康的同时，尊重个人权利和隐私的需求。这些法律法规为结核病感染控制打下坚定的基础。尽管法律法规框架彻底论述了结核病感染控制，但仍未评估其传播、实施和加强，也未评估其对公共健康的影响。

**结论:**

博茨瓦纳、南非和赞比亚的法律法规论述了世界卫生组织 (WHO) 关于结核病感染控制政策的原则。但是缺乏实施数据具有一定的限制性。未来研究应评估法律法规的实施和对公共健康的影响。

## Резюме

**Цель:**

Определить, согласованы ли законы и нормативные акты в Ботсване, Замбии и Южной Африке (эти три страны характеризуются высоким бременем туберкулеза и ВИЧ) с элементами политики Всемирной организации здравоохранения (ВОЗ) по борьбе с туберкулезной инфекцией.

**Методы:**

В ноябре 2015 года с помощью общедоступных национальных правовых баз данных был проведен интерактивный аналитический обзор законов и нормативных актов, согласующихся с шестью избранными элементами политики ВОЗ по борьбе с туберкулезной инфекцией в трех странах. Эти шесть элементов включали: (i) национальную политику и правовую базу; (ii) проектирование, строительство и использование лечебных учреждений; (iii) наблюдение за туберкулезом среди медицинских работников; (iv) права пациентов и медработников; (v) мониторинг мер по борьбе с инфекцией; (vi) исследования по соответствующей теме.

**Результаты:**

Было установлено, что эти шесть элементов должным образом учтены в законах и нормативных актах трех стран. Во всех трех странах существует требование об отчетности о случаях заболевания туберкулезом, а также о наблюдении за распространенностью этой болезни среди медработников. Нормативно-правовые базы каждой страны также учитывают необходимость в соблюдении прав личности и уважении неприкосновенности частной жизни при обеспечении охраны здоровья населения. Эти законы и нормативные акты создают прочную основу для борьбы с туберкулезной инфекцией. Хотя нормативно-правовые базы во многих аспектах содействуют борьбе с туберкулезной инфекцией, оценка распространения, реализации и контроля соблюдения их принципов, а также их влияния на общественное здравоохранение проведена не была.

**Вывод:**

Законы и нормативные акты в Ботсване, Замбии и Южной Африке согласованы со всеми шестью элементами политики ВОЗ по борьбе с туберкулезной инфекцией. Тем не менее недостатком является отсутствие данных по их исполнению. В дальнейшем следует провести исследование для оценки исполнения законов и нормативных актов, а также их влияния на общественное здравоохранение.

## Introduction

Tuberculosis is the leading cause of death among human immunodeficiency virus (HIV)-infected people in Africa,^1^^,^^2^ where 80% of HIV-positive tuberculosis cases and deaths occur.^3^ In sub-Saharan Africa, transmission of tuberculosis in health facilities (i.e. nosocomial transmission) has been identified as a major contributor to high tuberculosis rates.^4^ Although all health-care workers and patients are at risk of exposure to, and the acquisition of, tuberculosis,^5^^,^^6^ those with an HIV infection are at a particularly high risk. Substantial nosocomial transmission of multidrug-resistant and extensively drug-resistant tuberculosis has also been documented in Africa – these forms of the disease are far more costly and difficult to treat.^3^^,^^7^ Fortunately, the risk of nosocomial transmission can be minimized by a combination of managerial, administrative, environmental and personal protection measures, collectively referred to as tuberculosis infection control.^8^^,^^9^ Political commitment is critical for tuberculosis infection control and that commitment can be demonstrated by a country’s legislation,^8^ which may influence the practice of infection control. However, if we are to discern the impact of legislation on tuberculosis infection control, or on any other area of public health, we must first have information about the relevant laws – this may require a cross-disciplinary approach involving legal research and policy analysis.

Law, which has been defined as a government’s system of binding rules that order, permit, reward, forbid and punish behaviour,^10^ has been proven to have a positive effect on health in a wide range of public health areas, such as tobacco control, injury prevention and encouraging increased physical activity through urban design.^11^ The hierarchy of laws differs between countries but typically includes various types of legal instruments (Box 1). A country’s highest law – the constitution – sets out the broad structure of government as well as fundamental rights and duties. Legislation is a specific act or statute passed by the legislative or parliamentary branch of government to help implement the broad mandates of the constitution. For example, legislation can create a ministry of health to realize a constitutional right to health. A further step in operationalizing the law is the issuance of regulations by executive branch officials: for instance, ministers of health are often authorized by legislation to issue regulations. Law has long been central to public health, so much so that the Constitution of the World Health Organization (WHO) requires that: “Each Member shall communicate promptly to the Organization important laws, regulations, official reports and statistics pertaining to health which have been published in the State concerned”.^12^ However, in important areas of public health, such as tuberculosis infection control, the global health community lacks a sound understanding of the law’s effect on population health. As observed in the literature, “little national research, and even less cross-national comparative research, has been conducted to describe and analyse legislative approaches to tuberculosis control”.^13^ The discontinuation of WHO’s *International Digest of Health Legislation* makes it even more important that public health practitioners research, describe and analyse relevant public health laws.^14^

Box 1 **Hierarchy of laws and their relationship to public health practice**

**The constitution:**

• sets out rights, such as the right to health or health-care services;

• sets out duties, such as a state's obligation to realize the right to health;

• sets out civil liberties, which at times may conflict with public health objectives.

**Legislation:**

• builds on broader constitutional principles and aims by creating public health structures;

• creates entities such as a ministry of health and positions such as a minister of health;

• establishes requirements, objectives and processes and prohibits practices.

**Regulations:**

• are issued after legislation by a legislatively authorized entity or official;

• provide greater operational detail to guide additional policies, programmes and practice; for example, South African regulations authorizing conditional, temporary quarantine.

The aim of this review was to contribute to cross-national comparative research by describing and analysing legislative approaches to tuberculosis infection control practices in three countries in southern Africa with a high burden of HIV infection and tuberculosis: Botswana, South Africa and Zambia. Although the existence of laws and regulations does not ensure that they will be implemented,^15^ they provide the structural framework guiding tuberculosis infection control. Consequently, better understanding of existing laws in Africa that address tuberculosis infection control is a prerequisite to assessing their implementation and their effect on practice.^16^ The need for this understanding is urgent given the importance of controlling the transmission of tuberculosis in the continent.

## Methods

Two types of indicators have been proposed for assessing governance: rules-based and outcome-based indicators. Rules-based indicators “measure whether countries have appropriate policies, strategies and codified approaches for health system governance”, whereas outcome-based indicators “measure whether rules and procedures are being effectively implemented or enforced, based on the experience of relevant stakeholders”.^17^ We employed rules-based indicators to determine whether laws and regulations from three English-speaking countries in southern Africa^18^^–^^20^ address the six elements of the WHO policy on tuberculosis infection control listed in Box 2.^21^ Botswana, South Africa and Zambia were selected because they have a high burden of HIV-associated tuberculosis and high tuberculosis mortality. Further, more than 60% of tuberculosis patients in these three countries are HIV-positive and southern Africa has one of the highest tuberculosis incidence rates in the world.^22^^,^^23^ Consequently, our research is relevant to global efforts to control tuberculosis and HIV infection.

Box 2 **Do laws and regulations in Botswana, South Africa and Zambia address the WHO policy on tuberculosis infection control, 2015?**

**Element of the WHO policy on tuberculosis infection control ^21^**

1. National policy and legal framework for tuberculosis infection control: Yes, address explicitly.

2. Health facility design, construction, renovation and use: Yes, address explicitly.

3. Surveillance of tuberculosis disease among health workers: Yes, address explicitly.

4. Protection of patients’ and health workers’ rights and dignity: Yes, address explicitly.

5. Monitoring and evaluating tuberculosis infection control measures: Yes, address implicitly.

6. Enabling and conducting research to inform policy and practice: Yes, address implicitly.

WHO: World Health Organization.Note: Whether or not national laws and regulations address each element was categorized as “Yes, explicitly”, “Yes, implicitly” or “No”.

In November 2015, given the lack of a comprehensive global database of national health laws, we conducted an online desk review by searching publicly available national databases. The distinct nature of each database necessitated a slightly different approach to legal research in each country. For Botswana, the official Laws of Botswana database (updated until 2012)^24^ was searched for the terms “tuberculosis”, “infection”, “building control”, “health” and “research”. For South Africa, we browsed acts listed under the heading “Health” at the official South African Government website, which is continuously updated.^25^ For Zambia, the official Laws of Zambia database (updated until 1996) was used^26^ and supplemented by two unofficial legal databases containing more recent laws.^27^^,^^28^ Laws listed at these latter two websites were reviewed for relevance. Legal research was supplemented by a review of national tuberculosis infection control policies and guidelines made available to the authors by contacts in the countries concerned.

Finally, we used a checklist of six policy-related elements from the WHO policy on tuberculosis infection control as a benchmark for evaluating national policies.^21^ We selected the elements most relevant to national policy and limited the number to six to make the analysis of complex national laws and policies more manageable. Whether or not national laws and regulations addressed each element was categorized as “Yes, explicitly”, “Yes, implicitly” or “No”. “Yes, explicitly” was used if one or more laws found in our search at least partially addressed the selected element with specific reference to tuberculosis. “Yes, implicitly” was used if one or more laws were expressed in broad language that could be interpreted as addressing the selected element. “No” was used if no part of any law found could be interpreted as addressing the selected element.

## Results

We found that national laws and regulations in Botswana, South Africa and Zambia address all six selected elements of the WHO policy on tuberculosis infection control (Box 2). Each country’s legal and regulatory framework explicitly addresses elements 1 to 4, albeit in myriad ways. Elements 5 and 6 are implicitly addressed because national laws promote monitoring and evaluation and health research but use broad language that does not refer specifically to tuberculosis infection control.

### Botswana

In Botswana, several legislative acts and regulations are directly relevant to tuberculosis infection control (Box 3). The Public Health Act authorizes the isolation of persons certified to have a communicable disease on the order of a registered medical practitioner until such persons are determined to be free from infection or to no longer pose a danger to public health. The reporting of tuberculosis cases is also addressed by this Act, which lists tuberculosis as a notifiable disease and requires health officers to notify cases to the minister of health. Furthermore, Botswana’s Tuberculosis Infection Control Guidelines call for the screening of “all HCW (health-care workers) for tuberculosis disease and HIV infection routinely”. These Guidelines use mandatory language throughout (e.g. “must”), which may make it a binding policy document.

Box 3 **Elements of the WHO policy on tuberculosis infection control^21^ that are addressed in the laws and regulations of Botswana, 2015**

**1. National policy and legal framework for tuberculosis infection control**

Chapter 2 of the National Tuberculosis Infection Control Guidelines requires administrative controls, environmental controls and personal respiratory protection; Section 10 of the Public Health Act authorizes the isolation of persons with communicable diseases on the order of a medical practitioner, Section 16 allows for regulations to be made for the prevention of tuberculosis and other communicable diseases; Section 88 authorizes the minister of health to issue such regulations and Section 36 authorizes the isolation of persons entering Botswana who may have been exposed to, or are infected with, a communicable disease; Section 19 of the Nurses and Midwives Professional Ethics and Practice Regulations requires that midwives follow tuberculosis protocols and guidelines; and Section 22 of the Children in Need of Care Regulations requires compliance with health and building control standards for the establishment of child welfare institutions (i.e. orphanages) and Section 33 states that children cared for by institutions shall benefit from government programmes to control diseases, including tuberculosis.

**2. Health facility design, construction, renovation and use**

Section 4 of the Building Control Act allows for the regulation of the “ventilation of buildings”; Section 44 of the Public Health Act establishes that health officers have a duty to address “nuisances”, which, as detailed in Section 46, include a “premises or part thereof … which is or are likely to promote the spread of any disease”, a “premises which is so overcrowded as to be injurious or dangerous to the health of the inmates or is dilapidated or defective in lighting or ventilation”, or “any public or other building which is so situated, constructed, used, or kept as to be unsafe, or injurious or dangerous to health”; and the Development Control Code aims to regulate land use activity that “affects the quality of air …”, which might include ventilation.

**3. Surveillance of tuberculosis disease among health workers**

Section 5 of the Public Health Act lists tuberculosis as a notifiable disease and requires health officers to notify cases to the minister of health “in the prescribed form”; and Section 2.2.8 of the National Tuberculosis Infection Control Policy calls for action to “Screen all HCW (Health Care Workers) for tuberculosis disease and HIV infection routinely”.

**4. Protection of patients’ and health workers’ rights and dignity**

Health Professions Council (Professional Conduct) Regulation 21 requires health professionals to keep patients’ health information secret, although professionals may inform other health-care providers or regular close contacts of the patient who are at risk of acquiring a communicable disease.

**5. Monitoring and evaluating tuberculosis infection control measures**

Section 3 of the Public Health Act specifies that one function of the ministry of health is “to prepare and publish reports and statistics or other information relative to the public health”; and Section 28 of the Statistics Act charges Statistics Botswana with producing official statistics “derived from statistical censuses and surveys or the processing of administrative records”.

**6. Enabling and conducting research to inform policy and practice**

Section 3 of the Public Health Act specifies that one function of the ministry of health is “to promote or carry out researches and investigations in connection with the prevention and treatment of human diseases”; Section 7 of the Botswana Health Professions Act allows for the establishment of a Research and Publications Committee; and Section 3 of the Nurses and Midwives Professional Ethics and Practice Regulations includes the duty to “engage in research” in a list of duties of nurses or midwives.

HIV: human immunodeficiency virus; WHO: World Health Organization.

### South Africa

In South Africa, a complex assortment of acts, regulations and other policies governs tuberculosis infection control (Box 4). The highest law governing health in South Africa is the South African Constitution of 1996, Section 27 of which states in part, “everyone has the right to have access to: (a) health-care services”. The constitutional right to health-care services has been interpreted by the South African Constitutional Court in numerous cases but a review of case law is beyond the scope of the present paper.^30^^,^^31^

Box 4 **Elements of the WHO policy on tuberculosis infection control^21^ that are addressed in the laws and regulations of South Africa, 2015**

**1. National policy and legal framework for tuberculosis infection control**

The National Infection Prevention and Control Policy and Strategy, which applies to all public sector health-care facilities and providers, require the establishment of provincial, district and facility-level infection prevention and control committees (the national committee was previously established by the National Health Act), call for infection control risk assessments in all public facilities and set out the disciplinary, civil and criminal consequences of noncompliance with the policy and strategy and related guidelines; the Regulations Relating to Communicable Diseases and the Notification of Notifiable Medical Conditions authorize temporary quarantine of persons exposed to communicable diseases to prevent further spread (Section 2) and authorize the compulsory isolation and treatment of infected persons (Section 17); and Section 10 of the National Health Amendment Act created the Office of Health Standards Compliance to monitor and enforce the compliance of health establishments with national health system standards – in particular, to achieve certification, establishments must comply with National Core Standards for Health Establishments, which include the standards: “2.6.1 An Infection Prevention and Control Programme is in place to reduce health care-associated infections”; “2.6.2 Specific precautions are taken to prevent the spread of respiratory infections”; and “2.6.3 Standard precautions are applied to prevent health care-associated infections”, with relevant criteria being applied for each standard.

**2. Health facility design, construction, renovation and use**

Section 20 of the National Health Act establishes the rights of health-care personnel, which include ensuring that “every health establishment must implement measures to minimize… disease transmission”; Section 8 of the Occupational Health and Safety Act states: “Every employer shall provide and maintain, as far as is reasonably practicable, a working environment that is safe and without risk to the health of his employees” by “taking such steps as may be reasonably practicable to eliminate or mitigate any hazard or potential hazard to the safety or health of employees, before resorting to personal protective equipment”; Section 28 of the Act authorizes inspections and Section 43 authorizes the minister of manpower to regulate “the planning, layout, construction, use, alteration, repair, maintenance, or demolition of buildings”; and Regulations for Hazardous Biological Agents 1390 require the use of precautions to prevent standard and airborne transmission of tuberculosis, stating in particular: “Ideally place patients in a private room that has… monitored negative air pressure in relation to the surrounding areas, 6–12 air changes per hour, appropriate discharge of air outdoors or monitored high-efficiency filtration of room air before the air is circulated to other areas of the hospital. Where this is not possible, use a room with a simple extraction fan providing at least six air changes per hour, a room with an open window, and adequate ventilation.”

**3. Surveillance of tuberculosis disease among health workers**

Section 1 of the National Health Act defines “municipal health services” as services that include the “surveillance and prevention of communicable diseases, excluding immunisations”; Section 23 requires the National Health Council to advise the minister of health on policy concerning the “epidemiological surveillance and monitoring of national and provincial trends with regard to major diseases and risk factors for disease” and Section 90 authorizes the minister to make regulations regarding “communicable diseases” and “notifiable medical conditions”; Regulations Relating to Communicable Diseases and the Notification of Notifiable Medical Conditions include “All TB” and “Pulmonary Tuberculosis” as notifiable conditions;^29^ Section 43 of the Occupational Health and Safety Act authorizes the making of regulations for biological monitoring and medical surveillance of employees; the subsequently issued Regulations for Hazardous Biological Agents 1390 require medical surveillance of employees if “there is a reasonable likelihood that the disease or effect may occur under the particular conditions of his or her work and there are techniques such as pre-clinical biomarkers where appropriate for detecting sensitisation to allergens or an inflammatory response associated with exposure to diagnose indications of the disease or the effect as far as is reasonably practicable …”; and National Tuberculosis Management Guidelines call for medical surveillance of health-care workers, which includes periodical tuberculosis examinations.

**4. Protection of patients’ and health workers’ rights and dignity**

Section 9 of the Occupational Health and Safety Act states: “Every employer shall conduct his undertaking in such a manner as to ensure, as far as is reasonably practicable, that persons other than those in his employment who may be directly affected by his activities are not thereby exposed to hazards to their health or safety; and Section 7 of the National Health Act generally requires informed consent for health care and Section 14 requires the confidentiality of users’ health information unless “non-disclosure of the information represents a serious threat to public health”.

**5. Monitoring and evaluating tuberculosis infection control measures**

Section 21 of the National Health Act calls for the National Department of Health to “identify national health goals and priorities and monitor the progress of their implementation” and Section 25 calls for provincial departments to “provide services for the management, prevention and control of communicable and non-communicable diseases”.

**6. Enabling and conducting research to inform policy and practice**

Section 70 of the National Health Act states: “The National Health Research Committee must identify and advise the minister of health on health research priorities”; and Section 4 of the Medical Research Council Act enables health science research.

TB: tuberculosis; WHO: World Health Organization.

Further down the hierarchy of laws are several legislative acts that provide more specific direction on tuberculosis infection control. Section 10 of the National Health Amendment Act created the Office of Health Standards Compliance to monitor and enforce the compliance of all health establishments with national health system standards. Mandatory National Core Standards for Health Establishments, formulated by the Department of Health, set out three standards applicable to tuberculosis infection control: (i) “2.6.1 An Infection Prevention and Control Programme is in place to reduce health care-associated infections…”; (ii) “2.6.2 Specific precautions are taken to prevent the spread of respiratory infections”; and (iii) “2.6.3 Standard precautions are applied to prevent health care-associated infections”. Each of these standards has associated criteria. For example, one criterion used to determine the compliance of a facility with Standard 2.6.1 is whether “a qualified health professional is responsible for infection control”.

Requirements for appropriate health facility infrastructure and use are given by the Regulations for Hazardous Biological Agents 1390, which set out precautions to prevent standard and airborne transmission of tuberculosis. They describe an ideal scenario in which the patient is placed in a private room with negative air pressure and six to 12 air changes per hour and a scenario in which the ideal is not possible: for example, the patient may be placed in a room with a simple extractor fan, an open window and adequate ventilation.

Tuberculosis case notification is required under the Regulations Relating to Communicable Diseases and the Notification of Notifiable Medical Conditions and the Occupational Health and Safety Act allows for regulations to be made that require the medical surveillance of employees. Subsequently issued in 2001, Regulations for Hazardous Biological Agents 1390 require the medical surveillance of employees if a particular disease may occur at work and if techniques exist to “diagnose indications of the disease”. Given the evidence of nosocomial transmission of tuberculosis and the availability of diagnostic techniques, these regulations appear to require the surveillance of health-care workers for the disease. National Tuberculosis Management Guidelines reiterate the call for tuberculosis surveillance in health-care workers.

Patients’ rights and dignity are also addressed in the Occupational Health and Safety Act and the National Health Act. The former act mandates that all employers “ensure, as far as is reasonably practicable, that persons other than those in his employment… are not thereby exposed to hazards to their health or safety”. The National Health Act protects patients’ rights and balances the confidentiality of a patient’s health information against the need to disclose such information to prevent “a serious threat to public health”.

### Zambia

In Zambia, as in Botswana and South Africa, various laws and regulations govern tuberculosis infection control (Box 5). The Health Professions Act created the Health Professions Council of Zambia, which issued the Guidelines for Licensing of Health Facilities. The Guidelines require inspectors to evaluate a health facility’s compliance with maintenance, infection prevention and sanitation standards. For example, Standard 10 on infection prevention concerns the availability of infection prevention staff and of documents such as manuals and guidelines and Standard 11 on sanitation requires inspectors to assess the availability and use of personal protective equipment. Additionally, in 2012, the Health Professions Council issued the Health Professions (General) Regulations, which require self-assessment of infection prevention as part of the application process for the accreditation of health-care services.

Box 5 **Elements of the WHO policy on tuberculosis infection control^21^ that are addressed in the laws and regulations of Zambia, 2015**

**1. National policy and legal framework for tuberculosis infection control**

National Tuberculosis Infection Control Guidelines note the “urgent need for tuberculosis infection control in health care settings”, call for administrative (e.g. patient management), environmental (e.g. ventilation) and personal protective measures (e.g. respiratory protection and laboratory safety), recommend training for all health workers in tuberculosis infection control, appear to require health-worker safety measures and include a sample facility-level tuberculosis infection control plan; Section 4 of the Health Professions Act mandates the Health Professions Council to inspect and licence health facilities in compliance with infection prevention standards – the council issued Guidelines for Licensing of Health Facilities, including Standard 10 on infection prevention, which concerns the availability of infection prevention staff and of documents such as manuals and guidelines, and Standard 11 on sanitation, which requires inspectors to assess the availability and use of personal protective equipment; the Health Professions (General) Regulations require self-assessment of infection prevention as part of the application process for the accreditation of health-care services and define “infection prevention” as a “robust infection prevention programme that protects both the patients and the staff”; Section 15 of the Public Health Act authorizes: “Inspection of infected premises and examination of persons suspected to be suffering from infectious diseases”; and Public Health (Infectious Diseases) Regulation 59 requires teachers and students to be excluded from school for “tuberculosis of the lungs” until “production of a medical certificate of recovery and freedom from infection”.

**2. Health facility design, construction, renovation and use**

Public Health (Building) Regulation 44 requires the cross-ventilation of public buildings.

**3. Surveillance of tuberculosis disease among health workers**

Part III of the Public Health Act includes tuberculosis among notifiable diseases, Section 10 of the Act requires the reporting of notifiable disease cases to medical officers and Section 28 authorizes the minister of health to make regulations to prevent tuberculosis, including regulations on quarantine and isolation; Public Health (Infectious Diseases) Regulation 2 requires employers to report known cases of notifiable diseases among employees, Regulation 6 penalizes noncompliance and Regulation 7 requires medical officers to register notified cases of notifiable diseases, including tuberculosis; and, after registration, medical officers must use Form 2 of Public Health (Infectious Diseases) Regulation 10 to report weekly on all cases of, and deaths due to, notifiable diseases.

**4. Protection of patients’ and health workers’ rights and dignity**

Public Health (Infectious Diseases) Regulation 8 allows for the regulations to be enforced “without more hardship to any person than is necessary and unavoidable in the public interest”; and Section 4 of the Occupational Safety and Health Act establishes the Occupational Health and Safety Institute and Section 11 requires that: “An employer of ten or more persons at any workplace shall establish a health and safety committee” to develop standards, rules and procedures for occupational health and safety.

**5. Monitoring and evaluating tuberculosis infection control measures**

The National Health Strategic Plan calls for strengthening of tuberculosis infection control and of monitoring and evaluation; and the National HIV/AIDS/STI/TB Council Act supports monitoring and evaluation of these diseases.

**6. Enabling and conducting research to inform policy and practice**

The Tropical Diseases Research Centre Act created the Tropical Diseases Research Centre to promote research; the National HIV/AIDS/STI/TB Council Act charges the council with developing a national research agenda; and the National Health Research Act established an authority and ethics board to regulate health research.

AIDS: acquired immunodeficiency syndrome; HIV: human immunodeficiency virus; STI: sexually transmitted infection; TB: tuberculosis; WHO: World Health Organization.

Tuberculosis case-reporting is also required in Zambia. Under the Public Health Act, medical officers in the country must receive reports of all cases of notifiable disease, including tuberculosis. Also noteworthy is the requirement in Public Health (Infectious Diseases) Regulation 2 that all employers report cases of notifiable diseases among employees. In terms of record-keeping, Public Health (Infectious Diseases) Regulation 7 requires each medical officer to register notified cases of notifiable diseases, including tuberculosis. After cases are registered by the medical officer, he or she must use Form 2, given in Regulation 10, to report weekly on all cases of, and deaths due to, notifiable diseases.

Finally, with regard to the protection of patients’ and health workers’ rights, Zambia’s Public Health (Infectious Diseases) Regulation 8 seeks to strike a balance between respecting an individual’s rights and pursuing the public good “without more hardship to any person than is necessary and unavoidable in the public interest”.

## Discussion

Our search found numerous laws, regulations and other binding policies applicable to tuberculosis infection control in Botswana, South Africa and Zambia. This information adds to previous literature in other countries describing national tuberculosis legal frameworks and their importance to tuberculosis control.^32^^,^^33^

From our review, it is clear that all three countries have taken serious legal and regulatory steps to control infections, including tuberculosis. Although all the nations reviewed were found to have one or more laws or regulations that explicitly address each of the first four policy-related elements selected from the WHO policy on tuberculosis infection control (Box 2) and laws or regulations that implicitly address the last two, it should be noted that national frameworks differ. Botswana has an explicit policy to screen health workers routinely for tuberculosis and HIV. South Africa’s constitution sets out a universal right to health-care services and the country’s National Health Amendment Act helped realize that right by creating an Office of Health Standards Compliance. Zambia’s legal and regulatory framework is notable for the specificity with which it prescribes the reporting of tuberculosis cases and deaths, which involves the use of registers and of a special report form embedded in a regulation. One important similarity among Botswana’s, South Africa’s and Zambia’s legal and regulatory frameworks is their apparent requirement that tuberculosis surveillance be conducted among health-care workers: namely, health worker screening in Botswana, occupational health surveillance of employees in South Africa and an employer’s duty to report notifiable diseases in Zambia. Reporting of tuberculosis cases is also mandatory in all three nations.

Several of the laws and regulations we reviewed illustrate the need to balance the public interest against an individual’s civil liberties. South Africa’s National Health Act balances the confidentiality of a patient’s health information against an allowance for the disclosure of such information to prevent “a serious threat to public health”. In Zambia, Public Health (Infectious Diseases) Regulation 8 restricts individual hardship to that which is necessary and unavoidable, which may help ensure the government uses its authority to isolate and report people with communicable diseases in a manner that respects individual rights. This balancing of public interest and civil liberties is paramount in public health law, given the cost of excluding people from school, isolating them from social contacts and disclosing their disease status.

Limitations of this review include the lack of legal and regulatory documents in publicly available databases, the unknown reliability of the supplementary databases we used and possible overestimation of the extent to which the policy-related elements of the WHO policy on tuberculosis infection control were adequately addressed. An important limitation is the lack of any assessment of the extent to which applicable laws and regulations have been implemented or of their impact on public health. Nonbinding policies were not included in our review because they are less enforceable.

Our review has several implications for public health practice. The method we used enabled the systematic assessment of a variety of national laws and regulations across countries. Although we studied laws applicable to tuberculosis infection control, the method could readily be applied to assess health governance for other diseases or conditions. Clearly, if legal and regulatory frameworks do not adequately address tuberculosis infection control, it will be more difficult to improve infection control in practice or to benefit from the associated expected reduction in morbidity and mortality. This does not appear to be the case in the three countries assessed. However, even when national legal and regulatory frameworks appear adequate, one cannot assume they have an impact on tuberculosis infection control practice since laws require dissemination, training, implementation, compliance and enforcement to achieve their aims. Future research should use outcome-based indicators to assess the execution of national infection control laws in practice. For example, one could evaluate health workers’ knowledge of, attitudes to and practices concerning tuberculosis infection control laws in one or more of the countries we studied. Gaps in practice could thus be identified to improve implementation. Studies could also assess whether any variation in the degree of policy implementation across health facilities is associated with a variation in the level of tuberculosis infection control.

Our findings also have wider implications for global health. First, public health practitioners and their programmes would benefit from better understanding of existing domestic, legal and regulatory frameworks. Although one might think countries implementing global health programmes may lack appropriate laws on HIV, tuberculosis or any other area, whether or not they actually do should be systematically researched. Second, epidemiological and statistical analyses of the effect of structural factors on public health could be made more thorough by including as variables the findings of legal research (i.e. the laws identified and their specific characteristics). In these ways, systematic policy surveillance can contribute to scientific evaluations in public health.^34^^,^^35^

Laws are a concrete manifestation of leadership and governance that can have a positive impact on public health, especially when they are evidence-based and vigorously implemented or enforced.^36^ However, the extent of their effect on tuberculosis infection control in settings with a high prevalence of HIV infection has yet to be determined. The severity of the HIV–tuberculosis syndemic, particularly in southern Africa, means the need for such knowledge is urgent.
